# Spontaneous Hemodynamic Oscillations during Sleep Coordinate Directional CSF Flow in the Subarachnoid Space and Ventricles

**DOI:** 10.64898/2026.10.07.757422

**Published:** 2026-10-13

**Authors:** Danlei Chen, Fuyixue Wang, Amelia Strom, Hannah Yun, Jonathan R. Polimeni, Zijing Dong, Laura D. Lewis

## Abstract

During sleep, cerebrospinal fluid (CSF) flow is enhanced and thought to facilitate clearance of metabolic waste from the brain. Human imaging has shown that sleep is associated with large waves of bulk CSF flow in the fourth ventricle; however, the CSF flow dynamics around brain tissue are unknown. Here, we investigated how CSF flow is organized across the subarachnoid space, a key site both for fluid exchange across cortical surface and for transport out of the cranium. We used an MRI technology at 7 Tesla that simultaneously measures cortical hemodynamics and slow subarachnoid CSF flow and applied this during sleep. We found that spontaneous infraslow hemodynamic oscillations were coupled to spatially structured, directional CSF flow throughout both ventricular and subarachnoid compartments. Hemodynamic oscillations and fourth-ventricle bulk flow were both associated with brain-wide CSF flow at different phases, indicating that they reflect a single coordinated CSF movement cycle. Compared to cardiac pulsations, a canonical driver of CSF movement, hemodynamic-driven CSF flow showed a distinct directional pattern and was smaller in instantaneous amplitude but produced substantially greater cumulative displacement and thus greater potential for transport within an oscillation period. Together, these findings show that hemodynamic oscillations during sleep are coupled to temporally coordinated and directional CSF flow throughout the ventricles and subarachnoid space, suggesting that sleep-dependent neuronal activity drives fluid movement across the human brain in a distinct spatial pattern.

